# Editorial: Food, Nature, and Health: Dueling Epistemologies

**DOI:** 10.3389/fpubh.2016.00180

**Published:** 2016-08-25

**Authors:** Debbie L. Humphries, Molly D. Anderson, Padma Venkatasubramanian

**Affiliations:** ^1^Yale School of Public Health, New Haven, CT, USA; ^2^Middlebury College, Middlebury, VT, USA; ^3^Foundation for Revitalization of Local Health Traditions, Trans-disciplinary University, Bangalore, India

**Keywords:** knowledge generation, food systems, public health, epistemology, value systems

**The Editorial on the Research Topic**

**Food, Nature, and Health: Dueling Epistemologies**

Over the past two decades, the importance of addressing the complexity of the food system in ways that draw on new approaches to knowledge generation and new research paradigms has attracted significant attention (1, 2). This research topic explores the nexus of public health, food systems, food behavior, and food ways, focusing on belief systems, values, assumptions, and relationships to the food system, and approaches to understanding the impacts of food systems on human health. We propose some guidelines for consideration in designing research:

- Recognize that knowledge and practices in use by traditional societies for a long period of time probably have contemporary value (e.g., Payyappallimana and Venkatasubramanian).
- Initiate dialog between researchers in industrialized and traditional societies (e.g., Groot and Van’t Hooft) and compare practices in different kinds of societies (“Western” and “Eastern” in Dubé et al.).
- Question assumptions be aware that good intentions are no insurance against causing harm, and be open to the emergence of new information (Nair et al.). Assumptions include reliance on the scientific method as the only legitimate test of truth (3–6).
- Be aware that diagnoses focused on only part of the system are unlikely to effectively address underlying issues (Blay-Palmer, Keleman Saxena et al., and Jones et al.).

## Value of Traditional Practices

Ayurveda is a ~3000-year-old codified medical tradition of India still practiced today (Payyappallimana and Venkatasubramanian). Its science and practice are guided by universal principles including *panchamahabhutha* (five elements of Nature), *tridosa* (three humors), and *dravya guna sastra* (material science). Payyappallimana and Venkatasubramanian provide an overview of the Ayurvedic understanding of health, where *Svasthya* or health is seen as equilibrium at an individual level, varying from person to person depending on at least 10 intrinsic and extrinsic determinants of health. Equilibrium between the environment and self is a part of health. Transdisciplinary research on traditional medical systems that delves deeply into their epistemologies and principles may provide new perspectives on sustainable ways of living, in harmony with natural systems, embracing both human and planetary health.

## Cross-Cultural Dialog and Comparisons

Cross-cultural dialog between dairy farmers in the Netherlands and India is changing how farmers in both countries manage their herds (Groot and Van’t Hooft). The potential human health impacts of particular practices in dairy farming, including increasing emphasis on high productivity strains of cattle and increasing use of antibiotics to maintain animal health in high productivity settings, are a global concern (7). Groot and Van’t Hooft describe an ongoing international exchange and cross-cultural learning between dairy farmers, where farmers from the Netherlands and India spent 2 weeks visiting with their peers. The success of the India–Netherlands exchange led to the project being expanded to Ethiopia and Uganda in 2015.

Cross-cultural comparisons of perceptions can highlight value systems. Dubé et al. examine the relationship between perceptions of healthiness and taste of pulses in the United States and India. In Indian society, health and tastiness are strongly associated with natural products and traditional products. By contrast, in the United States, “traditional” foods are expected to taste better, but not necessarily to be healthy. The authors also explore how the amount of industrial processing influences perceptions of taste and health (Dubé et al.).

## Questioning Assumptions

Current recommendations to address micronutrient deficiencies around the globe focus on short-term supplementation, medium-term food fortification, and long-term dietary diversification, complemented by public health and disease control measures (8, 9). Nair et al. review the challenges involved in implementing the third strategy, long-term dietary diversification, including difficulties measuring dietary diversity, incomplete yet tantalizing evidence of the benefits of dietary diversity and co-benefits for outcomes ranging from reduced child stunting to improved gut health and income generation.

## Focusing on the Whole System

Food sovereignty is a unification of food, agriculture, human rights, and health agendas (Jones et al.). Jones et al. pose the question of whether the use of “ecologically sound and sustainable methods” of food production necessarily translate into better human health outcomes, and whether wider ownership of the agricultural or food system create gains in health and well-being (Jones et al.). They identify plausible linkages between food sovereignty and human health, but find that the empirical evidence in support of the hypothesis that increasing food sovereignty yields improvements to human health is limited.

In Bolivia, Keleman Saxena et al. describe how changes in rainfall induced by climate change are affecting food production. They argue for the importance of crosscutting studies that explicitly describe and explore linkages between climate/weather, environment, maintenance and use of agricultural biodiversity, cultural and food preferences of specific population groups, coping strategies that groups and individuals use to respond to climate and weather changes, and health outcomes that these factors synergistically generate. Such research may build on the robust body of existing studies in ecology, agronomy, and anthropology, but will require multidisciplinary research teams and complex research methods (Keleman Saxena et al.).

In Canada, Blay-Palmer compares the current market-based approach to children’s health in contrast to a rights-based approach, as directed by the Convention on Rights of the Child and other international treaties, and explores the social values underpinning current practices. The author argues that developed countries are neglecting their obligations under such international agreements, and that non-State actors are stepping up to fill the void through grassroots action. The author uses several case studies to illustrate this trend and concludes with an analysis of the implications of these findings in relation to the relevance of a rights-based approach to addressing food security for children in Canada (Blay-Palmer).

This research topic presents several ways to address food system-related health concerns that accommodate environmental constraints and have potential to meet public health goals more holistically than current approaches. Most of the papers speak to barriers − e.g., ideological, financial, and political economy − that have prevented greater open-mindedness in exploring such alternatives, and recommend additional research, emphasizing that the research must be directed in specific ways to uncover hidden and indigenous knowledge. These papers provide intriguing examples of how “blind spots” can be discerned and the acquired knowledge and training of scientists can be overcome.

## Author Contributions

DH, MA and PV drafted the editorial, participated in discussions about the ideas, and revised the final editorial.

## Conflict of Interest Statement

The authors declare that the research was conducted in the absence of any commercial or financial relationships that could be construed as a potential conflict of interest.
